# TRPC1 Channels Are Expressed in Pyramidal Neurons and in a Subset of Somatostatin Interneurons in the Rat Neocortex

**DOI:** 10.3389/fnana.2018.00015

**Published:** 2018-02-26

**Authors:** Juan R. Martinez-Galan, Ana Verdejo, Elena Caminos

**Affiliations:** Instituto de Investigación en Discapacidades Neurológicas, Facultad de Medicina, Universidad de Castilla-La Mancha, Albacete, Spain

**Keywords:** TRPC1, somatosensory cortex, pyramidal neurons, somatostatin interneurons, Martinotti cells

## Abstract

Disturbances in calcium homeostasis due to canonical transient receptor potential (TRPC) and/or store-operated calcium (SOC) channels can play a key role in a large number of brain disorders. TRPC channels are plasma membrane cation channels included in the transient receptor potential (TRP) superfamily. The most widely distributed member of the TRPC subfamily in the brain is TRPC1, which is frequently linked to group I metabotropic glutamate receptors (mGluRs) and to the components of SOC channels. Proposing TRPC/SOC channels as a therapeutic target in neurological diseases previously requires a detailed knowledge of the distribution of such molecules in the brain. The aim of our study was to analyze the neuroanatomical distribution of TRPC1 in the rat neocortex. By double- and triple-labeling and confocal microscopy, we tested the presence of TRPC1 by using a series of specific neurochemical markers. TRPC1 was abundant in SMI 32-positive pyramidal neurons, and in some glutamic acid decarboxylase 67 (GAD67) interneurons, but was lacking in glial fibrillary acidic protein (GFAP)-positive glial cells. In neurons it colocalized with postsynaptic marker MAP2 in cell bodies and apical dendritic trunks and it was virtually absent in synaptophysin-immunoreactive terminals. By using a panel of antibodies to classify interneurons, we identified the GABAergic interneurons that contained TRPC1. TRPC1 was lacking in basket and chandelier parvalbumin (PVALB) cells, and a very low percentage of calretinin (CALR) or calbindin (CALB) interneurons expressed TRPC1. Moreover, 63% of somatostatin (SST) expressing-cells and 37% of reelin-positive cells expressed TRPC1. All the SST/TRPC1 double-labeled cells, many of which were presumptive Martinotti cells (MC), were positive for reelin. The presence of TRPC1 in the somata and apical dendritic trunks of neocortical pyramidal cells suggests a role for this channel in sensory processing and synaptic plasticity. Conversely in SST/reelin interneurons, TRPC1 could modulate GABAergic transmission, which is responsible for shaping the coordinated activity of the pyramidal cells in the cortical network. In future studies, it would be relevant to investigate whether TRPC1 could be involved in the expression or processing of reelin in SST inhibitory interneurons.

## Introduction

Canonical transient receptor potential (TRPC) channels are plasma membrane, non-selective cation channels included in the transient receptor potential (TRP) superfamily of cation channels. In mammals, the TRP superfamily is divided into six subfamilies: TRPC, TRPV, TRPM, TRPA, TRPP and TRPML (for a review see Nilius et al., 2007; Venkatachalam and Montell, 2007). The TRPC subfamily consists of seven members (TRPC1 through to TRPC7). Each TRPC channel subunit is composed of six transmembrane domains and the assembly of four TRPC subunits forms functional channels. In the central nervous system (CNS), the most widely distributed member of the TRPC subfamily is TRPC1. TRPC1 is highly expressed in the hippocampus, amygdala, cerebellum (Strübing et al., 2001), substantia nigra (Martorana et al., 2006) and inferior colliculus (Valero et al., 2015). Whereas TRPC1 does not seem to form homomers in neurons, it forms functional heteromers with TRPC4 and TRPC5 (Strübing et al., 2001). Although other subunits, such as TRPC3, TRPC6 and TRPC7, have also been detected in the hippocampus or substantia nigra (Chung et al., 2006, 2007; Giampà et al., 2007), their contribution to form functional channels in the CNS still remains unclear.

In the brain, the Ca^2+^ influx, which is related to the opening of TRPC1, is frequently associated with the activation of group I metabotropic glutamate receptors (mGluRs). In the cerebellum, where this route has been widely studied, TRPC1 is localized in perisynaptic regions of dendritic spines, and is physically associated with mGluR1 (Kim et al., 2003). Moreover, Homer family proteins, which determine the spatial localization of group I mGluRs at the postsynaptic level, also facilitate the functional association of the inositol trisphosphate receptor (IP_3_R) with TRPC1 channels (Yuan et al., 2003). Despite ongoing discussion, some TRPC subfamily members, mainly TRPC1, can interact with the components of store-operated Ca^2+^ (SOC) channels (for a review see Salido et al., 2009; Lee et al., 2014). Generally, store-operated Ca^2+^ entry (SOCE) implies the activation of endoplasmic reticulum sensor protein STIM1 after Ca^2+^ stores depletion (Liou et al., 2007), and the formation of a plasma membrane pore that is permeable to extracellular Ca^2+^ through the polymerization of channel protein Orai1 (Prakriya et al., 2006).

TRPC1 participates in important neuronal processes related to synaptic transmission and plasticity (Bröker-Lai et al., 2017). In some brain disorders, TRPC1 plays a noteworthy role. While epileptiform burst firing, induced by group I mGluR agonists, reduces in TRPC1 knock-out mice (Phelan et al., 2012), TRPC1 overexpression can have a neuroprotective effect on hippocampal neurons (Wang et al., 2016). Cell calcium homeostasis disturbances, which are related to SOC channels, are also involved in neurological disorders such as Alzheimer’s (Sun et al., 2014), Huntington’s disease (Wu et al., 2016) and epilepsy (Steinbeck et al., 2011).

Proposing TRPC and/or SOC channels as a therapeutic target in neurological diseases previously requires having detailed knowledge of the neuroanatomical localization of such molecules in the brain. In the present study, we analyzed the distribution of TRPC1 in the neocortex of rat, namely in the somatosensory cortex, where a consistent and established neuronal classification is available (Markram et al., 2015). We observed that TRPC1 is exclusive of neurons, and is found abundantly in the somata and apical dendritic trunks of excitatory pyramidal neurons. We also demonstrated that TRPC1 is present in GABAergic interneurons. This channel predominates in a subpopulation of layer II/III somatostatin-(SST) containing neurons that always expresses reelin, a matrix extracellular protein which, in the adult cortex, play an important role in synaptic plasticity (Beffert et al., 2005; Chen et al., 2005; Groc et al., 2007) and in abundant neurological disorders (for a review see Folsom and Fatemi, 2013; Lussier et al., 2016).

## Materials and Methods

### Animals

Ten Wistar rats, ranging from postnatal day (P) 35 to P50, were used in this study (Charles River, Barcelona, Spain and Animal House of the Universidad de Castilla-La Mancha, Albacete, Spain). This study was carried out in accordance with Spanish (Real Decreto 53/2013) and European Union (2010/63/UE) regulations for the use and care of animals in research. The protocol was approved by the local (Welfare Animal Ethic Committee, Universidad de Castilla-La Mancha, ref. PR-2015-07-13) and National Committees (Consejería de Agricultura, Junta de Comunidades de Castilla-La Mancha) Spain.

### Primary Antibodies

Polyclonal anti-TRPC1 was purchased from Alomone Labs (#ACC-010; Jerusalem, Israel). TRPC1 was raised in rabbit against peptide QLYDKGYTSEQKDC, which corresponds to amino acid residues 557–571 of the intracellular domain of human TRPC1. The specifications of all the primary antibodies used in this study are shown in Table 1. The mouse monoclonal antibodies were the following: anti-neurofilament H non-phosphorylated (SMI 32) from Calbiochem (#NE1023, clone SMI 32, Bilerica, MA, USA), anti-glutamic acid decarboxylase 67 (GAD67) from Merck Millipore (#MAB5406, clone 1G10.02, Bilerica, MA, USA), anti-glial fibrillary acidic protein (GFAP) from Sigma (#G 3893, clone G-A-5, St. Louis, MO, USA), anti-microtubule-associated protein 2 (MAP2) from Sigma (# M 4403, clone HM-2), anti-synaptophysin from Sigma (#SAB4200544, clone SVP38), anti-parvalbumin (PVALB) from Sigma (#P 3088, clone PARV-19), anti-calretinin (CALR) from Swant (#6B3, Bellinzona, Switzerland), anti-calbindin D-28K (CALB) from Abcam (#ab82812; clone CB-955, Cambridge, UK) and anti-reelin from Merck Millipore (#MAB5364, clone G10). The goat polyclonal anti-SST was purchased from Santa Cruz Biotechnology (#sc7819).

**Table 1:** Primary antibodies.

Target protein	Antibody type/host species	Manufacturer	Catalog number/clone	Dilution
TRPC1	Polyclonal/rabbit	Alomone	ACC-010	1:200
SMI 32	Monoclonal/mouse	Calbiochem	NE1023/clone SMI 32	1:1000
GAD67	Monoclonal/mouse	Merck Millipore	MAB5406/clone 1G10.02	1:1000
GFAP	Monoclonal/mouse	Sigma	G 3893/clone G-A-5	1:500
MAP2	Monoclonal/mouse	Sigma	M 4403/clone HM-2	1:500
mGluR1/5	Monoclonal/mouse	Neuromab	75–116, clone N75/33	1:500
Synaptophysin	Monoclonal/mouse	Sigma	SAB4200544/clone SVP38	1:400
PVALB	Monoclonal/mouse	Sigma	P 3088/clone PARV-19	1:1000
CALR	Monoclonal/mouse	Swant	6B3	1:500
CALB	Monoclonal/mouse	Abcam	ab82812/clone CB-955	1:1000
SST	Polyclonal/goat	Santa Cruz Biothecnology	sc7819	1:2000
Reelin	Monoclonal/mouse	Merck Millipore	MAB5364/clone G10	1:1000

### Tissue Processing for Immunocytochemistry

Animals were deeply anesthetized with ketamine (100 mg/kg, Parke-Davis, Alcobendas, Spain) and xylazine (10 mg/kg, Dibapa, Barcelona, Spain). They were perfused through the left ventricle with 4% paraformaldehyde in 0.1 M phosphate buffer (PB), pH 7.4. Their brains were dissected out and post-fixed 4 h in fresh fixative at 4°C. Fifty micrometer-thick coronal sections that contained the primary somatosensory cortex were cut by a tissue slicer (VT1000S; Leica, Nussloch, Germany).

### Double Immunofluorescent Labeling

This procedure was used to test the presence of TRPC1 in different cell types and subcellular compartments. We previously verified the specificity of anti-TRPC1 by Western blot and by incubating sections in the appropriate blocking peptide provided by the manufacturer (Valero et al., 2015). The antibody was also tested by Sun et al. (2017) in the TRPC1^−/−^ mouse where no labeling was found. Anti-TRPC1 was combined with the following antibodies at the concentrations indicated in Table 1: anti-SMI 32 as a marker of pyramidal neurons; anti-GAD67 to evaluate the presence of TRPC1 in GABAergic cells; anti-GFAP to determine the presence of TRPC1 in astrocytes; anti-MAP2 to identify somata and dendrites; anti-synaptophysin to label presynaptic terminals. To evaluate the presence of TRPC1 in the neocortical interneurons, several markers were used: anti-PVALB, anti-CALR, anti-CALB, anti-SST and anti-reelin.

Free-floating sections were pretreated with 4% BSA, 3% normal goat serum and 0.1% Triton X-100 in phosphate buffered saline (PBS) at RT for 30 min, and were then incubated with anti-TRPC1 and the corresponding primary antibody at the dilution indicated in Table 1, at room temperature (RT) for 24 h. All the following steps were carried out in the dark at RT. After several rinses in PBS, sections were incubated for 60 min by using the following secondary antibodies: (1) Biotinylated goat anti-rabbit IgGs (Vector Laboratories Inc., Burlingame, CA, USA), diluted at 1:100 when the labeling for TRPC1 was combined with the protein targets raised in mouse. (2) Biotinylated rabbit anti-goat IgGs (Vector Laboratories Incorporation), at 1:200 and Cy5-conjugated anti-rabbit IgGs (Amersham), diluted at 1:200, for SST/TRPC1 double labeling experiments.

After rinsing, sections were incubated in Alexa 488-conjugated streptavidin for 60 min (Life Technologies, Gran Island, NY, USA) diluted at 1:1000 and (1) Cy5-conjugated anti-mouse IgGs (Amersham) at 1:200; or (2) Cy5-conjugated anti-rabbit IgGs (Amersham), diluted at 1:200. After rinsing, sections were incubated in Hoechst 33258 (Sigma), diluted at 1 μg/ml to counterstain cell nuclei and to distinguish the layers of the cortex. After rinsing in PBS, sections were mounted and coverslipped with Cytoseal (Stephens Scientific, Wayne, NJ, USA).

### Triple Immunofluorescent Labeling

To test the presence of reelin in the SST/TRPC1 double-labeled cells, anti-TRPC1 (polyclonal, rabbit), anti-SST (polyclonal, goat) and anti-reelin (monoclonal, mouse) were used. Similar procedures, as described in the anterior subsection, were followed with the following cocktail of secondary antibodies: biotinylated rabbit anti-goat IgG together with Alexa 488-conjugated streptavidin, Cy3-conjugated anti-mouse IgGs (Amersham), at 1:200, and Cy5-conjugated anti-rabbit IgGs.

### Confocal Microscopy, Quantification and Statistical Analysis

Immunofluorescent sections were examined under a Zeiss LSM 710 laser scanning confocal microscope (Zeiss, Jenna, Germany) of continuous spectral detection, equipped with excitation laser lines at 405, 458, 488, 514, 561 and 633 nm. They were visualized through the following objectives: 20× dry objective (Zeiss, NA = 0, 8), 40× oil-immersion objective (Zeiss, NA = 1, 3) and 63× oil-immersion objective (Zeiss, NA = 1, 4). Images were recorded through separate channels for Hoechst 33258 (abs: 352 nm, em: 461 nm), Alexa 488 (abs: 498 nm, em: 520 nm), Cy3 (abs: 553 nm, em: 568 nm) and Cy5 (Cy5 abs: 650 nm, em: 670 nm). By using the same confocal settings, no specific labeling was found in the control sections incubated in the absence of primary antibodies. All the images shown in the figures are 2D single plane images obtained from 3D confocal image stacks (10–15 μm-thick).

To estimate the percentages of colocalization of TRPC1 with interneuronal markers PVALB, CALR, CALB, SST and reelin, manual cell counting was performed in at least 10 confocal images from different sections per neuronal marker from cortical layers I, II/III and IV/VI, obtained from a minimum of two animals at different rostrocaudal levels of the S1 cortex (Table 2). For this purpose, we used the tile scan function software of the confocal microscope, which assembles single images acquired at high resolution with a 20× or 40× objective to form one large superimage of the specimen that contains all the layers of the cortex.

**Table 2:** Number of cells immunoreactive to canonical transient receptor potential 1 (TRPC1) among total cells positive for individual chemical markers in cortical layers of the somatosensory cortex.

Neuronal marker (n, s)	TRPC1-marker double labeled cells/marker-ir cells			
	Layer I	Layer II/III	Layer IV/VI	Total
PVALB (2, 10)	-	-	-	-
CALR (2, 10)	-	17/65	9/74	26/139
CB (2, 10)	-	18/74	9/127	27/201
SST (3, 20)	-	104/144	62/119	166/263
Reelin (2, 20)	6/36	67/144	27/89	100/269

*Animals (n) and sections (s) used for the analysis*.

Data were analyzed using GraphPad Prism (version, 5.01) software. The statistical analysis of proportions of TRPC1 immunoreactivity across different interneuron populations (CALR, CALB, SST and reelin) was accomplished by chi-square test. The null hypothesis states that TRPC1 expression is independent of the cell type. Chi-square test was also applied to compare the distribution of TRPC1 between the layer II/III and IV/VI in each of the populations studied (Table 3). The null hypothesis assumes that TRPC1 expression in a specific population of interneurons is independent of the neocortical layers.

**Table 3:** Statistical analysis of the proportion of TRPC1 immunolabeling between layers II/III and IV/VI.

Marker	Chi-square	*p* value df = 1
CALR	4.45	*p* < 0.05
CALB	11.95	*p* < 0.001
SST	11.33	*p* < 0.001
Reelin	5.99	*P* < 0.05

## Results

### Distribution of TRPC1 in the Cellular Subtypes of the Neocortex

**Figure 1:**
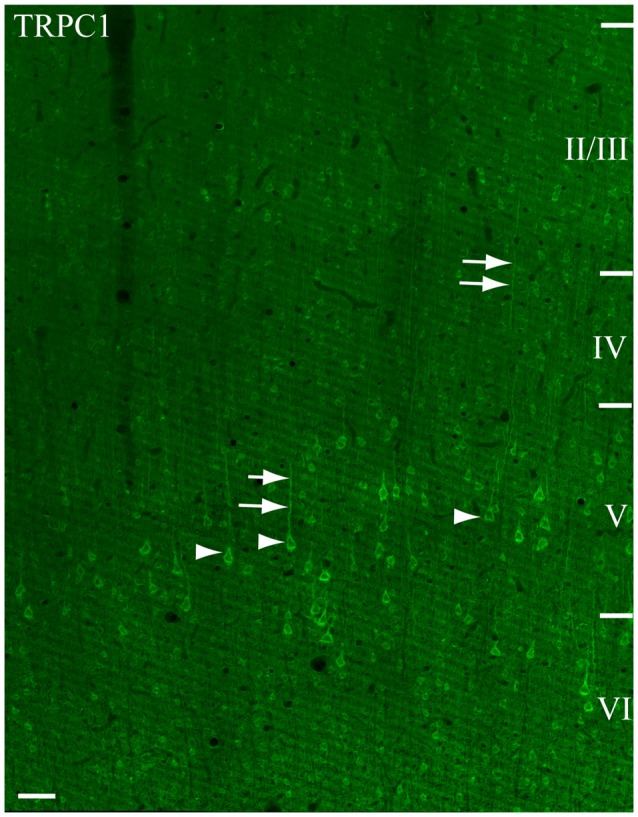
Immunofluorescence for Canonical transient receptor potential 1 (TRPC1) in the primary somatosensory cortex. The confocal mosaic single plane image of an S1 cortex coronal section shows the distribution of TRPC1. TRPC1 is expressed through all the neocortex layers. The cell bodies (arrowheads) and apical shafts (arrows) of pyramidal neurons are strongly immunoreactive to TRPC1. Cortical layers are indicated with roman numerals. Scale bar: 50 μm.

**Figure 2:**
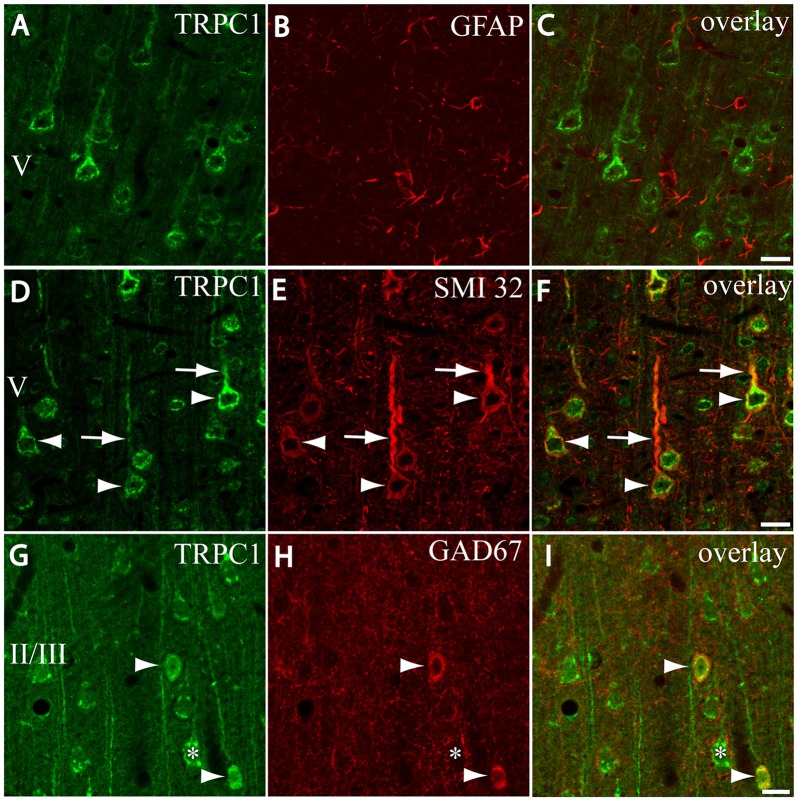
Distribution of TRPC1 in the cell populations of the primary somatosensory cortex. **(A–I)** Confocal images show the double labeling of TRPC1 (Alexa 488, green) with glial fibrillary acidic protein (GFAP), SMI32 or glutamic acid decarboxylase 67 (GAD67; all visualized with Cy5, red). **(A–C)** No colocalization of TRPC1 with GFAP was observed. **(D–F)** Many TRPC1-ir cells expressed SMI32 at layer V of the neocortex. Double labeling was found in neuronal somata (arrowheads) and apical shafts (arrows). **(G–I)** TRPC1 occasionally colocalized with GAD67-ir neurons (arrowheads). The GABAergic terminal surrounding somata (asterisk) and dendritic shafts of pyramidal TRPC1-ir neurons, unstained for GAD67, are shown. The cortical layer is indicated with roman numerals. Scale bar: 20 μm.

We used single immunofluorescence to study the pattern of TRPC1 distribution in the somatosensory cortex. A representative tile scan of adjacent images, acquired at high resolution, is shown in Figure 1. Although TRPC1 was expressed at all the layers of the cortex, it was clearly visible in abundant cell bodies (arrows) and apical shafts (arrowheads) of the pyramidal neurons of layer V (Figure 1). Double immunofluorescence labeling was performed to study the specific localization of TRPC1 in different cell types (Figure 2).

First, we evaluated the presence of TRPC1 in astrocytes by using astroglial marker GFAP. No colocalization of both TRPC1 and astroglial marker GFAP was observed (Figures 2A–C). Whereas abundant cell somata and apical shafts were labeled for TRPC1 in cortical layer V, astrocytes and GFAP-positive glial processes were clearly negative.

Next we were interested in confirming the presence of TRPC1 in neurons. For this purpose, we used SMI32, an antibody against a neurofilament that is expressed by cortical neurons, particularly the subcortical projecting neurons of layer V (Voelker et al., 2004). The arrowheads in Figure 2D show representative layer V neurons positive to TRPC1, which were immunoreactive to SMI32 (Figure 2E and the merged image in Figure 2F). All the SMI32-immunoreactive (SMI32-ir) cell somata were immunostained for TRPC1. The double-labeled apical dendritic shafts of the pyramidal neurons are indicated by arrows.

Afterward, we evaluated the presence of TRPC1 in the cortical interneurons, which constitute approximately 20%–30% of the remaining neurons in the neocortex (for a review see Markram et al., 2004). As most are GABAergic, we used an antibody against GAD67, the enzyme that participates in the synthesis of GABA. The images in Figures 2G–I show a region of layer II/III where some TRPC1-positive neurons colocalized with GAD67 (arrows). The asterisk denotes a soma immunostained for TRPC1 that was negative to GAD67, and corresponded to a pyramidal neuron. GAD67 labeling was also observed in the GABAergic terminals onto the soma and apical shaft of pyramidal cells. Therefore, our results indicated that TRPC1 was absent in astrocytes and expressed in cortical excitatory pyramidal and inhibitory interneurons.

### Subcellular Distribution of TRPC1 in the Neuronal Compartments of the Neocortex

**Figure 3:**
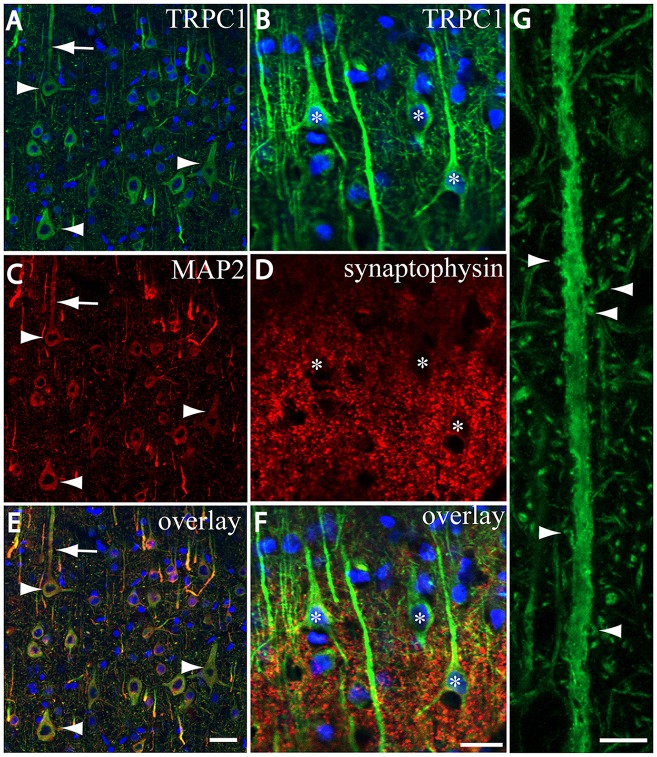
TRPC1 is located in postsynaptic compartments. **(A–G)** Confocal images, all obtained from neocortical layer V, show the double labeling of TRPC1 (Alexa 488, green) with microtubule-associated protein 2 (MAP2) and synaptophysin (all visualized with Cy5, red). **(A,C,I)** All the TRPC-ir somata (arrowheads) and abundant TRPC1-ir processes were positive to MAP2. Note the apical shaft pointed by the arrow. **(B,D,F)** TRPC1-ir somata (asterisks) and apical shafts rarely colocalized with synaptophysin terminals. **(G)** High magnification of an apical shaft of a layer V pyramidal neuron shows some TRPC1-ir dendritic appendages (arrowheads) corresponding to dendritic spines. Nuclei are counterstained in blue **(A–F)**. Scale bar: 20 μm in **(A–F)**; 5 μM in **(G)**.

We tested whether the pattern of subcellular distribution of TRPC1 in neocortical neurons was coincident with the data reported in the literature for Purkinje neurons, where it is selectively associated with the dendritic spines of Purkinje cells (Kim et al., 2003). We used double immunofluorescence labeling of TRPC1, together with dendritic and somatic marker MAP2 or presynaptic marker synaptophysin. All the TRPC-ir somata (the arrowheads in Figures 3A,C,E), and many TRPC1-ir processes were positive to MAP2. These processes correspond to dendrites and apical shafts of pyramidal neurons (arrow in Figures 3A,C,E). In addition, the colocalization of synaptophysin-ir puncta with TRPC1 was scarce and restricted to some terminals found over TRPC1-ir somata and dendritic trunks. TRPC1 somata are indicated by asterisks in Figures 3B,D,F. Figure 3G shows a high magnification of an apical shaft of a layer V pyramidal neuron with TRPC1-ir tiny dendritic appendages (arrowheads) corresponding to dendritic spines.

### Distribution of TRPC1 in Neocortical Interneurons

**Figure 4:**
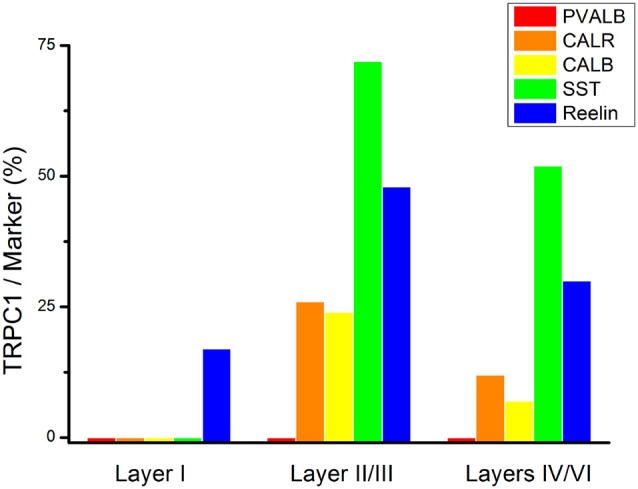
Colocalization of TRPC1 with the interneuronal markers in the rat somatosensory cortex. Percentages of a specific neuronal marker that was also positive to TRPC1 analyzed per layer I, II/III and IV/VI. TRPC1 expression was more prominent in the somatostatin (SST) interneurons. Note that the percentage of TRPC1 colocalization with calretinin (CALR), calbindin (CALB), SST and Reelin was the uppermost at layer II/III.

To study the distribution of TRPC1 in GABAergic interneurons, we used the double immunofluorescence of TRPC1 with different molecular markers of interneurons. Table 2 shows the number of neurons that contained a specific marker which were immunoreactive to TRPC1 of all the cells that contained the marker. It also provides the total number of animals and sections analyzed in this study. Table 3 shows the differences in the distribution of TRPC1 between layer II/III and IV/IV in each of specific populations. Figure 4 shows the percentages of the specific neuronal marker that was also positive to TRPC1 analyzed per layers I, II/III and IV/VI.

**Figure 5:**
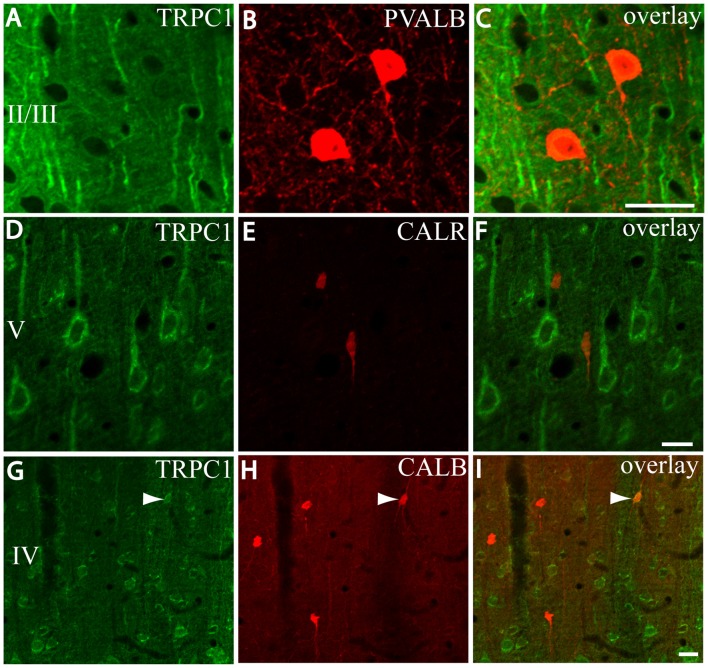
Distribution of TRPC1 in the calcium-binding proteins immunoreactive interneurons. **(A–I)** Confocal images show the double labeling of TRPC1 (Alexa 488, green) with PVALB, CALR or CALB (all visualized with Cy5, red). **(A–C)** TRPC1 never colocalized with PVALB. **(D–I)** The presence of TRPC1 in CALR or CALB-ir neurons was scarce. **(D–F)** Two CALR-ir neurons, which lacked TRPC1, are shown. **(G–I)** Three CALB-ir neurons and one double-labeled neuron (arrowhead) are shown. The cortical layer is indicated with roman numerals. Scale bar: 20 μm.

First, we evaluated the colocalization of TRPC1 with calcium-binding proteins PVALB, CALR and CALB. We started the analysis with PVALB, which is present in approximately 50% of GABAergic neurons (Uematsu et al., 2008). No colocalization was found between PVALB and TRPC1, nor in any of the layers of the studied somatosensory cortex. Figures 5A–C show two representative PVALB-ir neurons from layers II/III that lacked TRPC1 expression. Regarding CALR, the colocalization with TRPC1 was scarce (13% of all the CALR-ir cells). Figures 5D–F show two CALR-ir neurons from layer V that lacked TRPC1. We observed more colocalization at layers II/III (26%) than at layers IV/VI (12%). The colocalization between CALB and TRPC1 was also modest (13%), scarce at layers IV/VI (7%) and slightly more abundant at layers II/III (24%). Figures 5G–I show three CALB-ir neurons from layer IV that lacked TRPC1, and a cell immunoreactive to CALB and TRPC1 (arrowhead).

**Figure 6:**
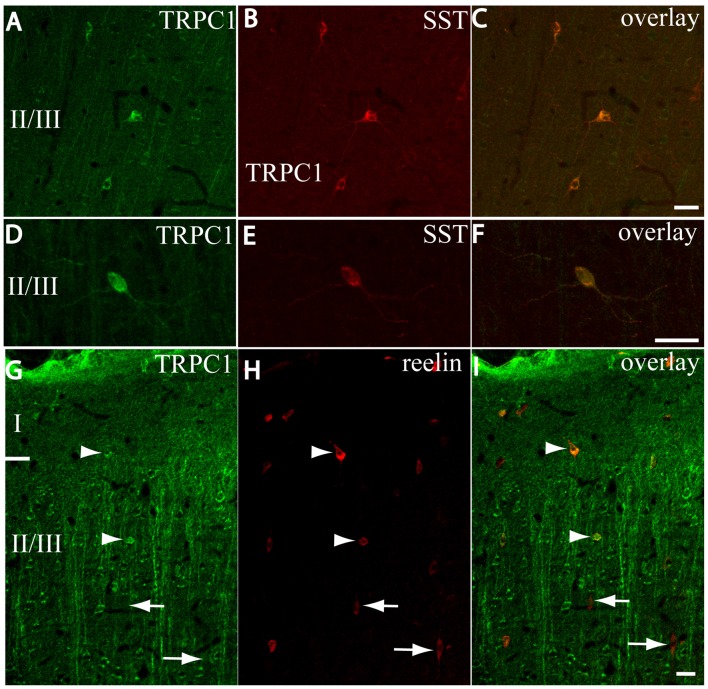
Distribution of TRPC1 in the SST or reelin-ir interneurons. **(A–I)** Confocal images show the double labeling of TRPC1 (green) with SST or reelin (red). **(A–C)** Example of the TRPC1-expressing cells at layer II/III immunoreactive for SST. TRPC1 was frequently expressed in the neurons that contained SST. **(D–F)** A neuron immunopositive to TRPC1 and SST with ovoid-shaped soma and multipolar dendritic arborization is shown. **(G–I)** Two reelin-ir cells of the supragranular layers immunolabeled to TRPC1 (arrowheads) and two TRPC1-ir cells that lacked reelin (arrows) are shown. Scale bar: 20 μm.

After the PVALB-expressing neurons, the SST-containing group constituted the second most abundant population of neocortical interneurons (Uematsu et al., 2008). The degree of colocalization of SST with TRPC1 was 62% of the SST-ir neurons, the highest among all the studied markers. Figures 6A–C show some TRPC1 expressing cells at layer II/III, which were immunoreactive for SST. Colocalization was more frequent at layers II/III (72%) than in the granular and infragranular layers (52%). In some of these double-labeled cells, ovoid-shaped soma and multipolar dendrite arborization can be observed (Figures 6D–F). Finally, we studied extracellular matrix protein reelin, which is found in different proportions in the interneurons of the somatosensory cortex (Pohlkamp et al., 2014). The colocalization of reelin with TRPC1 was 37% and with the following distribution: 17% at layer I, 48% at layer II/III and 30% at layer IV/VI. A higher degree of colocalization was also found for upper layers. Figures 6G–I show two reelin-ir cells of the supragranular layers immunolabeled to TRPC1 (arrowheads) and two TRPC1-ir cells that lacked reelin (arrows).

Statistical analysis (chi-square test) demonstrated that TRPC1 distribution among the different studied markers (CALR, CALB, SST and reelin) was not homogeneous. Differences were statistically significant in layer II/III (chi-square = 62.99; *p* < 0.001) and layer IV/VI (chi-square = 73.98; *p* < 0, 001). Moreover, for any of the studied markers, the expression of TRPC1 was significantly higher in the layer II/III than in the layer IV/VI (Table 3).

### Reelin Expression in the Double-Labeling SST/TRPC1-containing Neurons

**Figure 7:**
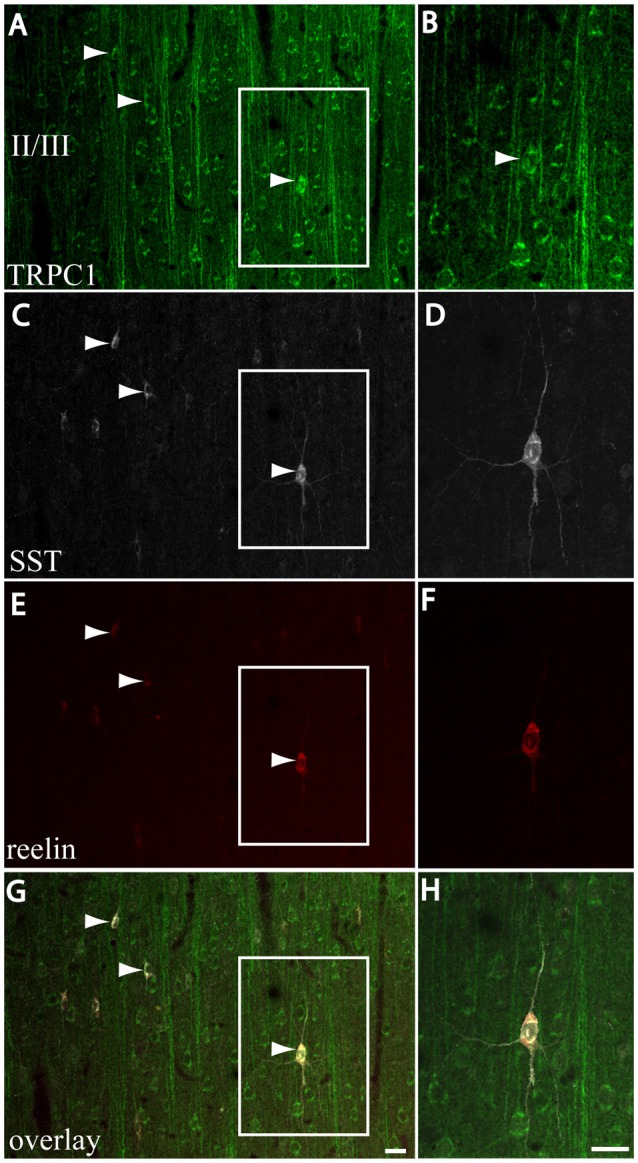
Expression of reelin in the SST/TRPC1 interneurons. **(A–H)** Confocal images show the triple labeling of TRPC1 (green) with SST (white) and reelin (red). The images on the left column show that all the TRPC1/SST cells colocalized reelin; three are indicated by arrowheads **(A,C,E,G)**. A high magnification of a triple labeled neuron is shown on the right column **(B,D,F,H)**. Scale bar: 20 μm.

We next studied whether reelin was expressed in the SST/TRPC1 subpopulation of interneurons by triple-labeling immunofluorescence. Figures 7A,C,E,G show three double-labeled cells to SST and TRPC1 (arrowheads) from layer II/III, which were immunoreactive for reelin. Furthermore, all the SST/TRPC1 interneurons (*n* = 52) were immunoreactive to reelin. A high magnification of the triple-labeled interneuron in the rectangle is illustrated in Figures 7B,D,F,H.

## Discussion

Here we studied TRPC1 distribution in the somatosensory neocortex of rat. Our objective was to determine the cell populations in which TRPC1 is expressed. We used double- and triple-labeling experiments to colocalize TRPC1 with a variety of neuronal markers that are widely described in the literature. The major findings of this study were: (1) TRPC1 is absent in astrocytes, but is abundantly expressed in pyramidal cells and in a subset of interneurons; (2) TRPC1 is found in the somato-dendritic compartment of neocortical cells; (3) in interneurons, TRPC1 is expressed mainly in SST cells, and in some CALB, CALR and reelin cells; and (4) the SST neurons that express TRPC1 always contain reelin.

### TRPC1 Is Expressed in Excitatory and Inhibitory Neocortical Neurons

We first checked the presence of TRPC1 in astrocytes. As expected, no presence of TRPC1 was found in cortical astrocytes. Although the expression of TRPC1 and its relation with SOCE have been demonstrated in cultured cortical astrocytes (Golovina, 2005; Malarkey et al., 2008; Reyes et al., 2013), we found no colocalization with GFAP in cortical tissue, which agrees with previous data reported by other authors in the rat substantia nigra (Martorana et al., 2006), and also with our results in the inferior colliculus (Valero et al., 2015). In fact, while SOCE can be consistently recorded in cultured astrocytes and blocked by Zn^2+^ after metabotropic receptor stimulation, this mechanism of Ca^2+^ influx is not observed in astrocytes *in situ* where the plateau phase is lacking (Pivneva et al., 2008). Our results support the notion that functional and ultrastructural differences exist in the Ca^2+^ intracellular stores between cultured and *in situ* astrocytes.

The next step was to elucidate the distribution of TRPC1 in neocortical neurons. TRPC1 has been observed in pyramidal neurons of the hippocampus (Strübing et al., 2001; von Bohlen Und Halbach et al., 2005; Chung et al., 2006), However, we found not detailed study about what TRPC1 distribution is like in the neocortex. Here we describe the presence of TRPC1 in cortical pyramidal neurons by immunofluorescent labeling. The location, neuronal morphology and coexpression of SMI32 support this affirmation. Nevertheless, TRPC1 is not exclusive of pyramidal cells. Although not uniformly distributed, TRPC1 is also found in some GAD67-ir cells.

### Postsynaptic Location of TRPC1

All TRPC1-ir cell bodies and many TRPC1-ir processes were positive to dendritic marker MAP2. Furthermore, TRPC1-ir tiny appendages from apical shafts also indicate a localization of TRPC1 in dendritic spines. In contrast, synaptophysin-ir terminals rarely contained TRPC1. The somato-dendritic distribution of TRPC1 coincides with that found in other brain areas, such as the hippocampus (Strübing et al., 2001), substantia nigra (Martorana et al., 2006), cerebellum (Kim et al., 2003) and inferior colliculus (Valero et al., 2015). In these regions, a TRP-like Ca^2+^ extracellular influx can be evoked after the pharmacological activation of group I mGluRs (Gee et al., 2003; Kim et al., 2003; Tozzi et al., 2003; Valero et al., 2015). At the ultrastructural level, group I mGluRs are generally located in the postsynaptic membrane, namely in dendrites and dendritic spines (Lujan et al., 1996). In the cerebellum, a physical and functional interaction between TRPC and group I mGluRs exists (Kim et al., 2003), which is facilitated by Homer family proteins, which also facilitate the mechanical link between IP3R and TRPC1 (Yuan et al., 2003). Although the association between TRPC1 and group I mGluRs seems very likely to be in the neocortex, it cannot be ruled out that TRPC1 could also be activated by cholinergic inputs through muscarinic receptors, whose presence is also relevant in pyramidal cells (Rahman and Berger, 2011; Dasari et al., 2017) and interneurons (Kawaguchi, 1997).

In the striatum, TRPC1 forms a complex with STIM1 that regulates the survival of dopaminergic neurons (Sun et al., 2017). TRPC1 distribution in the neocortex is also similar to that described for STIM1, one of the main regulators of SOC channels. STIM1 is found mainly in apical dendrites and neuronal somata of mouse cortical layer V (Klejman et al., 2009). Whether TRPC1 can form functional complexes in the neocortex with STIM/Orai proteins remains unknown to date. Interestingly, a channel composed of STIM2/Orai2, together with TRPC6, has been described in hippocampal CA1 neurons by Zhang et al. (2016). This channel, which is responsible for the maintenance of the postsynaptic dendritic spines, is involved in Alzheimer’s disease in experimental models. In cultured neurons it is known that STIM1/Orai1 interact with anchorage proteins in the spine apparatus (Korkotian et al., 2014), and play a functional role in spine formation (Korkotian et al., 2017). Experiments performed at optical and ultrastructural levels in brain histological sections are necessary to examine *in situ* whether TRPC1 can also form complexes with STIM1/Orai1. Nevertheless, the poor efficiency of the available antibodies to STIM and Orai in fixed tissue is a main limitation to fulfill this goal.

### TRPC1 Is Expressed Mainly in SST Interneurons

We tested the distribution of TRPC1 in GABAergic cells through a series of commonly used markers of interneurons. The classification of the neocortical interneurons has been a topic of debate for many years. However, morphological, neurochemical and physiological criteria have been standardized to reach a consensus about a classification of cortical neurons (Ascoli et al., 2008; DeFelipe et al., 2013; Markram et al., 2015). First we studied the distribution of TRPC1 in PVALB-expressing neurons. We did not observe any TRPC1 expression in PVALB-ir cells at any cortical layer. PVALB is a calcium-binding protein present in 40%–50% of GABAergic interneurons (Gonchar et al., 2008; Uematsu et al., 2008; Xu et al., 2010) and is commonly expressed in fast spiking chandelier and large basket cells (for a review see DeFelipe, 1997; Markram et al., 2004; Vitalis and Rossier, 2011). Regarding CALR, the colocalization with TRPC1 was modest, but somewhat more abundant at upper layers. CALR is a calcium-binding found in double bouquet, bipolar and bitufted cells. When we studied the colocalization of TRPC1 with CALB, the pattern was the same as that observed for CALR, and with similar proportions and at a higher degree of colocalization with TRPC1 at supragranular layers. CALB is present in some basket and dendrite-targeting cells, such as double bouquet and bitufted cells. The colocalization of TRPC1 with SST was more important. It was the highest among all the studied markers (63%) and was once again higher in supragranular layers (72%) than in lower layers (52%). After PVALB neurons, SST neurons constitute the second most abundant population of neocortical interneurons, with about 20%–30% of GABAergic cells (Gonchar et al., 2008; Uematsu et al., 2008; Xu et al., 2010). Both PVALB and SST neurons are medial ganglionic eminence- (MGE) derived neurons (Fogarty et al., 2007), but are not overlapping populations (Xu et al., 2010). SST is found mainly in Martinotti cells (MC), whose axons innervate the distal dendrites of pyramidal cells at layer I. The presence of TRPC1 in MC is consistent with a high expression of mGluR1 (Cauli et al., 2000; Stinehelfer et al., 2000; Cosgrove and Maccaferri, 2012). The colateral axons from pyramidal cells would be the main source of glutamatergic input to these cells (Adesnik et al., 2012). Muscarinic cholinergic receptors, whose activation causes persistent firing in MC (Fanselow et al., 2008), could be other molecular partners that interact with TRPC1. In the somatosensory cortex, MC are important in context-dependent sensory processing. Namely at layer II/III, MC are suppressed by vasoactive intestinal peptide (VIP) interneurons after whisking activation (Muñoz et al., 2017). Interestingly we found the highest percentage of SST/TRPC1 colocalization at layer II/III. SST is also distributed in a limited fraction of basket and bitufted cells (for a review see Liguz-Lecznar et al., 2016). Unlike MC, layer IV non Martinotti SST interneurons target mainly PVALB fast spiking cells instead of pyramidal cells (Xu et al., 2013). Finally, we analyzed the colocalization of TRPC1 with reelin. Reelin is expressed in a variety of GABAeregic interneurons in different proportions, including SST neurons (Alcántara et al., 1998; Pesold et al., 1999; Pohlkamp et al., 2014). TRPC1 was expressed in many reelin neurons (37%), and mainly at upper cortical layers, which coincides with the other markers (48%). As shown, a high proportion of SST MGE derived neurons also contained reelin (Miyoshi et al., 2010). We wondered whether SST/TRPC1 neurons also contained reelin. We demonstrated that all the SST/TRPC1 neurons were reelin-ir, which indicates that a characteristic of SST/reelin MGE-derived neurons should be TRPC1 expression. Therefore, we demonstrated that TRPC1 is enriched in SST/reelin cells, mainly at supragranular layers, and is probably represented by layer II/III MC, and restricted to other populations of dendrite-targeting cells capable of expressing different combinations of SST, CALB, CALR, reelin, but not PVALB.

In conclusion, our data indicate that TRPC1 is present in cortical pyramidal cells, likely into the dendritic spine, which suggests possible involvement in synaptic plasticity, and can even be linked to SOC channels. In contrast in supragranular SST/reelin interneurons, calcium dynamics, due to TRPC1 activation through mGluR1 receptor activation and/or basal forebrain cholinergic modulation, could play an active role in the inhibition of pyramidal cell firing during coordinated cortical activity in the absence of whisker stimulation. Further studies are required to investigate whether TRPC1 could be involved in reelin expression/processing in SST inhibitory interneurons.

## Author Contributions

All the authors have made substantive contributions to article for having credit as authors. Individual tasks of the authors were as follows: conception of the work (JRM-G), design of the experiments (JRM-G), carrying out the experiments (JRM-G and AV), acquisition of confocal images (JRM-G and AV), quantitative analysis (EC) and interpretation of data (all authors). Drafting the work (JRM-G and EC); revising it critically for important intellectual content (all authors). Final approval of the latest version (all authors).

## Conflict of Interest Statement

The authors declare that the research was conducted in the absence of any commercial or financial relationships that could be construed as a potential conflict of interest.
